# Health communication in and out of public health emergencies: to persuade or to inform?

**DOI:** 10.1186/s12961-022-00828-z

**Published:** 2022-03-05

**Authors:** Andrew D. Oxman, Atle Fretheim, Simon Lewin, Signe Flottorp, Claire Glenton, Arnfinn Helleve, Didrik Frimann Vestrheim, Bjørn Gunnar Iversen, Sarah E. Rosenbaum

**Affiliations:** 1 Centre for Epidemic Interventions Research, Norwegian Institute of Public Health, Skøyen, Postboks 222, 0213 Oslo, Norway; 2 Faculty of Health Sciences, Oslo Metropolitan University, Oslo, Norway; 3 Health Systems Research Unit, South African Medical Research Council, Cape Town, South Africa; 4 Department of General Practice, Institute of Health and Society, University of Oslo, Oslo, Norway; 5 Centre for Evaluation of Public Health Measures, Norwegian Institute of Public Health, Oslo, Norway

**Keywords:** Evidence-informed health policy, Health communication, Risk communication, Health promotion, Infodemic, Health education, Health information, Persuasion, Ethics

## Abstract

Much health communication during the COVID-19 pandemic has been designed to persuade people more than to inform them. For example, messages like “masks save lives” are intended to compel people to wear face masks, not to enable them to make an informed decision about whether to wear a face mask or to understand the justification for a mask mandate. Both persuading people and informing them are reasonable goals for health communication. However, those goals can sometimes be in conflict. In this article, we discuss potential conflicts between seeking to persuade or to inform people, the use of spin to persuade people, the ethics of persuasion, and implications for health communication in the context of the pandemic and generally. Decisions to persuade people rather than enable them to make an informed choice may be justified, but the basis for those decisions should be transparent and the evidence should not be distorted. We suggest nine principles to guide decisions by health authorities about whether to try to persuade people.

## Potential conflicts between seeking to persuade or seeking to inform

During the pandemic, governments and health authorities have recommended or mandated infection prevention and control measures, including social distancing, face masks, travel restrictions, self-isolation, quarantines, lockdowns and vaccination. Implementation of these measures has ranged from simply informing the public, to eliminating people’s ability to choose.

Public messaging about these control measures has changed as the pandemic has evolved [1]. Changes may have reflected evolving research evidence and shifting expert opinions. However, justifications have not always been shared candidly in communication by governments or health authorities to the public [2–4]. Additionally, researchers may have hyped the certainty and potential of their research in order to promote it [5]. As a result, the public has sometimes experienced COVID-19 messages from these authorities as untruthful and inconsistent. Thus, those messages may have exacerbated rather than reduced confusion from the tsunami of information that accompanied the pandemic.

### Increasing compliance through persuasion

Authorities seeking to maximize compliance may design their communication to persuade people to follow recommended or mandated control measures. However, messages designed to persuade can limit people’s ability to make informed choices and may erode public trust in authorities, which in turn can negatively impact compliance. There is evidence that public trust in government increased compliance with stringent government restrictions in both authoritarian and democratic countries [6]. Furthermore, research needed to reduce uncertainties (such as randomized trials measuring the effects of closing schools) can be difficult to conduct in an environment where those uncertainties are not acknowledged publicly.

### Supporting informed choices providing options and the pros and cons of those options

Conversely, health authorities aiming to enable people to make informed choices (or to be transparent about the reasons for a mandate) are more likely to include what is known about the pros and cons of interventions and the reasons for recommendations or policies [7]. This approach respects the rights of individuals to be informed and enables participation in public debate. More candid communication might also make policy changes seem less arbitrary and help preserve people’s trust in health authorities [8, 9]. However, this approach could reduce compliance. For example, people may be less likely to wear face masks if they perceive them to be ineffective, and communication of uncertainty might reduce the perception of effectiveness [10]. It could also increase inequities, if some people are less likely to have access to candid information, to understand it or to be able to use it as intended [11].

Sometimes the goals of persuading and informing people are in conflict [11, 12]. This dilemma, brought into sharp contrast by the COVID-19 pandemic, exists for all types of health authorities, including public health professionals and organizations, other healthcare professionals and organizations, researchers and scientific organizations.

### Changing people’s behaviour through spin

One way to influence people to behave in a desired way is to emphasize the advantages of the desired behaviour and ignore or downplay any disadvantages or uncertainties (Table 1). This is sometimes referred to as “spin” or “hype”. It can be done intentionally or unintentionally. Spin can be found in the scientific literature, where reporting practices distort the interpretation of results and mislead readers so that results are viewed in a more favourable light [13–15]. It can be found in news reports [16], advertisements used to promote the purchase of health products [17], and in public health messages [18]. Spin is manipulative when it ignores or misinforms about events or alternatives.

**Table 1:** Ways of spinning information to influence people

Factors that can affect a decision	Spin to influence people to behave in a desired way
The effects of behaving in the desired way compared to other options	Emphasize or exaggerate the benefits of behaving in the desired way
	Ignore or downplay the harms or undesirable effects of behaving in the desired way
	Ignore or downplay uncertainty about the benefits, and emphasize or exaggerate uncertainty about the harms
	Neglect to consider or point out that people may weigh desirable and undesirable outcomes differently
	Assume or imply that the desirable effects far outweigh the undesirable effects
Costs of behaving in the desired way compared to other options	Ignore or downplay the costs and emphasize or exaggerate the savings of behaving in the desired way
	Ignore or downplay uncertainty about the savings and emphasize or exaggerate uncertainty about the costs
	Ignore, assume or imply the intervention is cost-effective, and ignore uncertainty
Alternatives to the desired option	Misinform or leave out information about relevant alternatives

Governments may limit the extent to which spin can be used by industry. For example, the European Union (EU) Directive 2005/29/EC on unfair commercial practices prohibits misleading and aggressive advertising [19]. This includes advertising that significantly limits the consumer’s ability to make an informed decision. *The Blue Guide on Advertising and Promotion of Medicines in the UK* states, “An advertisement must present information which is factually correct, and those facts should not be exaggerated in any way” [20]. However, such regulations do not apply to health authorities. For example, the Food and Drug Administration (FDA) in the United States required producers of Relenza and Tamiflu to state in the package labels that these drugs had not been proven to reduce complications of influenza. However, the Centers for Disease Control and Prevention (CDC) in the United States could claim that these drugs reduced complications and saved lives [21].

Strategies that can be used to influence people to behave in a desired way, either intentionally or unintentionally, include using words and hyperbolic or alarming language without presenting numbers [7, 22], presenting risk ratios for benefits and absolute effects for harms [7, 23], leaving out the denominator [24], arousing fear or a sense of urgency [25, 26], using narratives [27, 28], and using expert sources to support otherwise unsubstantiated claims [29].

Information designed to enable informed choices should systematically and transparently summarize the evidence and other considerations in relation to factors that might influence a decision, such as the factors listed in Table 1 [30]. When there is compelling evidence that the advantages far outweigh the disadvantages, the difference between persuading and informing people may be smaller. For example, the advantages of vaccines for measles, mumps and rubella clearly outweigh the disadvantages [31]. Parents want balanced information about the benefits and harms of childhood vaccination [32], and providing them with clear, concise, evidence-based information may both build trust and persuade them to consider the evidence when deciding.

However, the more closely balanced the advantages and disadvantages are and the greater the uncertainty, the more likely it is that there will be a difference between persuading and informing people. For example, for some women it is not clear whether the advantages of breast cancer screening outweigh the disadvantages. Consequently, communication to increase uptake differs substantially from information designed to enable informed decisions [33].

### The ethics of persuasion

**Fig. 1:**
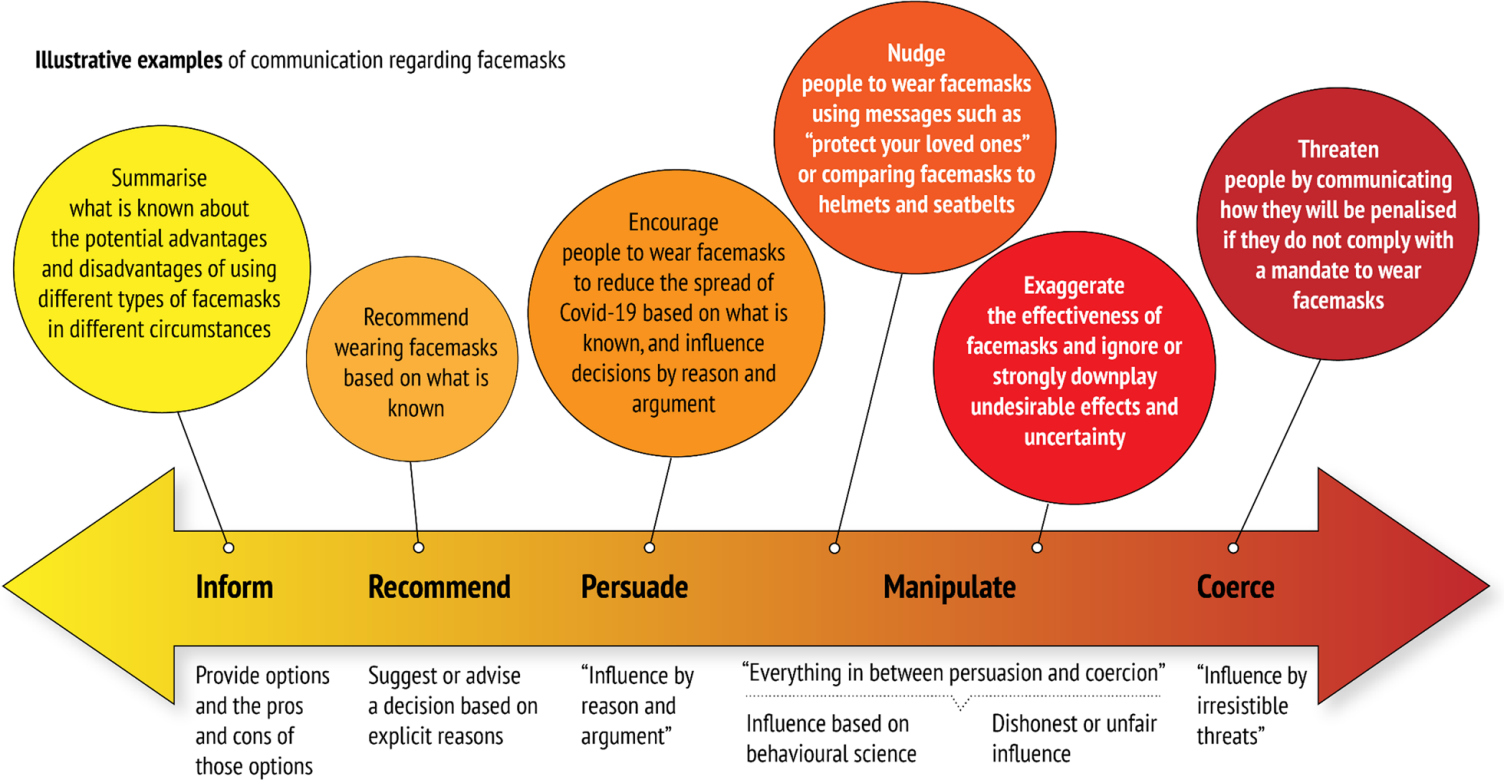
A continuum from information to coercion

When considering the ethics of information designed to persuade, it is helpful to recognize a continuum from information to coercion (Fig. 1). Persuasion, manipulation and coercion are the predominant categorizations of various types of influence in bioethics, although not all ethicists agree with these categories [18, 34]. Importantly, it has been argued that “manipulation” includes many forms of influence and needs conceptual refinement and ethical analysis to address the use of behavioural science to influence health behaviours. Also, these words may be confusing, since they can have different meanings and connotations in other contexts. Nonetheless, the distinctions or spectrum can be helpful when considering the ethics of persuasion.

#### Information that respects individual autonomy but considers collective burden

Information designed to inform people builds on a basic principle of respect for people’s autonomy [35]. Some autonomous choices that people make entail risks, such as riding a motorcycle. In societies that value autonomy, such choices are respected if they do not harm other people or create undue collective burden. In the context of a pandemic, many choices that people make can harm others and add to the collective burden (for example, on healthcare systems). Consequently, health authorities have frequently advocated policies that restrict autonomy and governments have implemented restrictive measures.

#### Information that is not manipulative

Information designed to influence people’s behaviour does not necessarily infringe on their autonomy, but it can if the information is manipulative [18]. Spin is manipulative if it promotes disinformation or withholds important information to direct people’s choices. For example, withholding important information about a well-documented, serious vaccine side effect that may lead people to choose not to be vaccinated or not to vaccinate their children would be manipulative, even if there is compelling evidence that the benefits far outweigh the harms. Providing information designed to arouse fear or other emotions, such as guilt or urgency, can also be manipulative. For example, during the COVID-19 pandemic, informing people about the gravity of the situation has been used to motivate people to adhere to control measures. People should be made aware of the seriousness of the situation so that they can make informed choices. However, exaggeration can exacerbate fear, anger and anxiety unnecessarily [36].

It can be argued that people’s choices are not truly “autonomous” when they are unknowingly shaped by their environment or by misinformation provided by actors with special interests [11], for example the food industry [37]. In addition, people do not always rationally weigh their options, and decisions are often affected by cognitive biases [38]. However, this alone does not justify manipulation of information or people’s emotions by health authorities or governments.

#### Information that is honest and transparent

When authorities deliberately design information to be persuasive or “manipulative” (using behavioural science), there is an underlying assumption that they know what problems should be addressed, what preferences and goals people have, and what is best for people and the community. If these assumptions are well founded, authorities may be justified in recommending, persuading or even restricting people’s behaviour, despite some disagreement. For example, seat belt laws, traffic regulations and information to promote adherence to those are widely accepted as well founded in many countries, although not everyone agrees.

However, when there are important uncertainties or disagreements, not being honest and transparent can inhibit research and perpetuate practices that are wasteful and may be harmful. This includes uncertainty or disagreements about social, economic and other consequences not directly related to health. Moreover, one key asset for obtaining public health goals, trust, may be undermined if health authorities are not transparent or perceived to be honest by the public. Changes in policies because of changes in the evidence are likely to be more acceptable to the public if the authorities were transparent about the uncertainties of the evidence when the original policy was made.

#### Information that does not “blame the victim”

Health information that is designed to influence people’s behaviour can also result in victim-blaming and stigmatization. For example, well-intended information campaigns to reduce obesity and the health consequences of obesity may have contributed to blaming, shaming and stigmatizing obese people [39]. Health communication about HIV and AIDS that used threats or scare tactics contributed to stigmatization [40]. The use of threats or scare tactics during the COVID-19 pandemic also may have contributed to stigmatization [36].

#### Information that is “actionable”

Not everyone wants to be informed or to make their own decisions about many of the behaviours that affect health [41]. Most people want clear, actionable messages, and for some people that is sufficient. For example, when there is a high COVID-19 infection rate, a recommendation to “wear face masks when it is not possible to maintain social distancing” is a clear actionable message. Not everyone is interested in the justification for such recommendations. Nonetheless, the justification should be reasonable, should be communicated transparently and should be available to anyone who is interested [46, 47].

#### Information that is developed through systematic, transparent and evidence-informed processes

It may be justified to design messages to persuade people to adhere to such recommendations. Health authorities who make decisions about what to recommend and whether to use persuasive messages should use systematic procedures informed by the best available evidence [30]. Systematic procedures should be used to decide how to communicate important recommendations, as well as for deciding what to recommend [42, 43]. Systematic procedures and transparency do not guarantee reasonable decisions any more than they guarantee that the results and interpretation of research are valid. Nonetheless, they can help to ensure accountability and reasonableness.

### When is it justified to persuade people to change their behaviour?

**Fig. 2:**
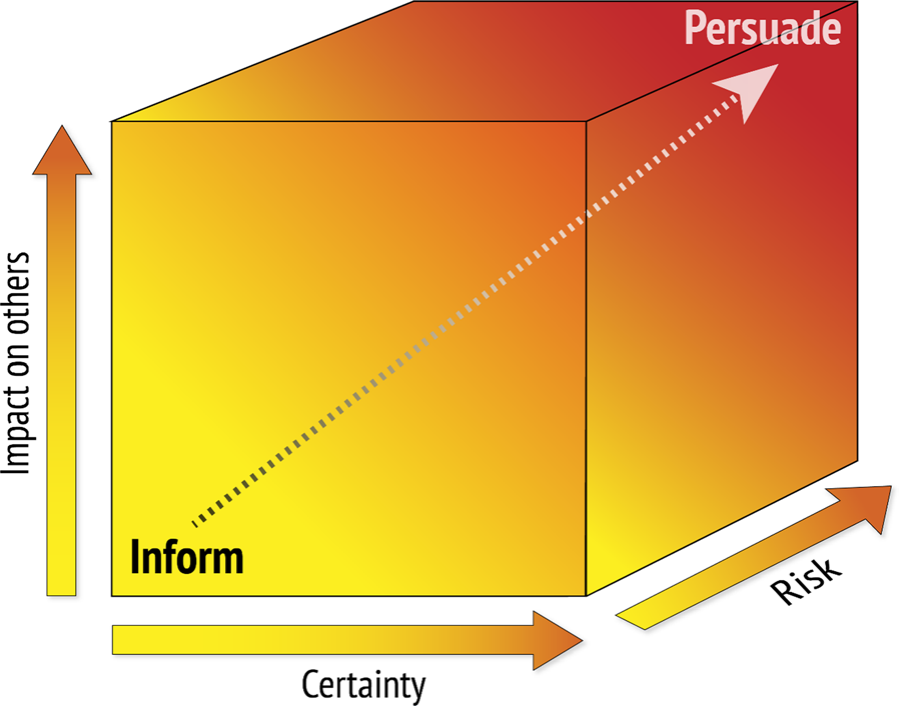
Factors underlying the justification for persuading people to change their behaviour

Generally, the more uncertainty there is about the balance between the advantages and disadvantages of a behaviour, the less likely it is that it is justified to try to persuade people to behave in that way (Fig. 2). On the other hand, the greater the potential impacts of a behaviour are on others (e.g. transmission of infectious diseases or drunk driving), the more likely it is that persuasion is justified [44]. Similarly, the greater the risk, the more likely it is that persuasion is justified.

### Communication in the context of public health emergencies

Health authorities who communicate to the public in the context of health emergencies, such as the COVID-19 pandemic, must take account of ethical considerations and the extent to which persuasion is justified. The extent of uncertainty combined with the need to respond urgently may limit the ability of health authorities and governments to use systematic and transparent processes to decide what to recommend and how to communicate recommendations. However, they can be prepared for emergencies by having established systematic processes for rapidly reviewing the evidence and making recommendations and policy decisions that are informed by existing evidence [45, 46], and by implementing evidence-based guidance for communicating risk and evidence [1, 7, 47]. They also can have in place processes for generating evidence to address important uncertainties [48].

Another way in which they can be prepared is by fostering critical thinking [49, 50]. The COVID-19 pandemic has been accompanied by an “infodemic”—an overabundance of information, some accurate and some not. By fostering critical thinking skills, health authorities and governments can help to reduce people’s susceptibility to misinformation and enhance their ability to recognize and use reliable information. Currently many people lack those skills [49–51].

### Principles to guide decisions by health authorities about whether to persuade

Health authorities and others responsible for communicating health information to the public should reflect carefully on the purpose of the information they communicate to the public—whether it is intended primarily to persuade people or to inform them. This should include consideration of long-term consequences as well as immediate effects on behaviour. For example, authorities who aim to persuade the public and therefore downplay uncertainty about the risk of side effects of a vaccine may increase uptake. However, if side effects are discovered over time, this could undermine trust and people’s willingness to be vaccinated in the future.

Before designing information to influence people’s behaviour in a specific direction, health authorities should be confident that the potential advantages outweigh the potential disadvantages and that most well-informed people would agree with their justification for wanting to persuade people. Deciding how to influence people parallels how to make recommendations based on evidence of variable quality [30, 52–54]. Generally, when there is low confidence in the evidence, strong recommendations and persuasion are not warranted. However, there are circumstances where a strong recommendation or persuasion is warranted despite important uncertainties [52–54]. Principles that can help guide decisions about when it is justifiable for health authorities to try to persuade people to behave in a certain way are summarized in Table 2. Answering the questions in Table 2 requires evidence, interpretation of the evidence, and judgements. Having in place an efficient system for summarizing the evidence, involving stakeholders and making transparent judgements can help to ensure that decisions and recommendations by public health authorities do more good than harm. Experience with such systems for deciding what to do or recommend can facilitate making similarly transparent judgements about how to communicate those decisions and whether to persuade people.

**Table 2:** Principles to guide decisions by health authorities about whether to persuade

Principles	Questions	Explanations
Evidence	What is known about the potential impacts of the behaviour?	Decisions about what to recommend should be based on the best available scientific evidence about the effects of the targeted behaviour, based on up-to-date systematic reviews whenever possible [58]. This is not always possible in the context of public health emergencies, but such reviews can be done rapidly [59], and experience using a structured approach to make and justify recommendations outside of emergencies can make it easier to do this in an emergency
	What is known about the potential impacts of the communication strategy?	Decisions about how to inform or persuade people should also be informed by the best available scientific evidence. It is possible to systematically review this evidence outside of the context of emergencies, so that it is readily available as evidence-based guidance [1, 6, 50], and experience using a structured approach outside of emergencies can also make it easier to do this in an emergency
Participation	Does the message reflect the values of those affected?	Decisions about whether and how to persuade depend on judgements about how much people value the potential benefits and harms. Stakeholders—those who are affected by the decision—should be involved in those decisions. For this to be practical in the context of emergencies, it is likely necessary to have established effective mechanisms to facilitate participation or participation in planning when not in the emergency [55–59]
Equity	Are the potential impacts of the message on different populations fair?	A decision to persuade (or not to persuade) should not affect segments of the population, particularly disadvantaged ones, unfairly. The benefits, harms and burden should be distributed fairly
Transparency	What is the justification for the message?	The justification for a decision to persuade should be transparent and readily available to the public. This should include the criteria used to make the decision, the judgements that were made for each criterion, and the basis for the judgements [30]
Precaution	Is there a credible threat of serious harm that warrants an urgent message?	In response to urgent and credible threats of serious harm, proportionate precautions should be taken. This principle is especially relevant in the context of public health emergencies. This is a complex principle that requires judgements about the urgency of a threat, the credibility of the threat, the likelihood and seriousness of the potential harms, and the potential benefits and harms of the intervention [60]. When the precautionary principle is applied, it should include evaluation to address important uncertainties, so far as possible [48]
Proportionality	Is the message appropriate for the level of risk?	The proportionality principle is used in a variety of contexts and has been defined in different ways [61]. The general idea underlying the principle is that responses to threats or risks should be appropriate for the level of risk and not excessive
Flexibility	Is the message tailored to key target audiences and their contexts and can it be modified as new information becomes available?	Messages should be tailored to address important cultural, socioeconomic and language differences. Attention should be paid to minority groups, their ability to access and understand messages, and their ability to act on messages. Choice of communication channels (e.g. websites, social media, mass media) should also be tailored to targeted audiences. Thresholds (e.g. for COVID-19 infection rates) and other reasons for changing the message should be communicated as clearly as possible, and it should be possible to reconsider and change messages and how they are communicated in response to changing conditions or new evidence
Testing	Has the message and how it is communicated been tested?	Important public health messages—whether they are designed to inform or persuade—and ways of communicating them should be tested with people from key targeted audiences, including minority groups, to ensure that they are correctly understood and helpful [42, 43]
Uncertainty	Are there important uncertainties about the impacts of the message?	Important uncertainties should be identified. When there are important uncertainties, the impacts of decisions should be evaluated as rigorously as possible

**Fig. 3:**
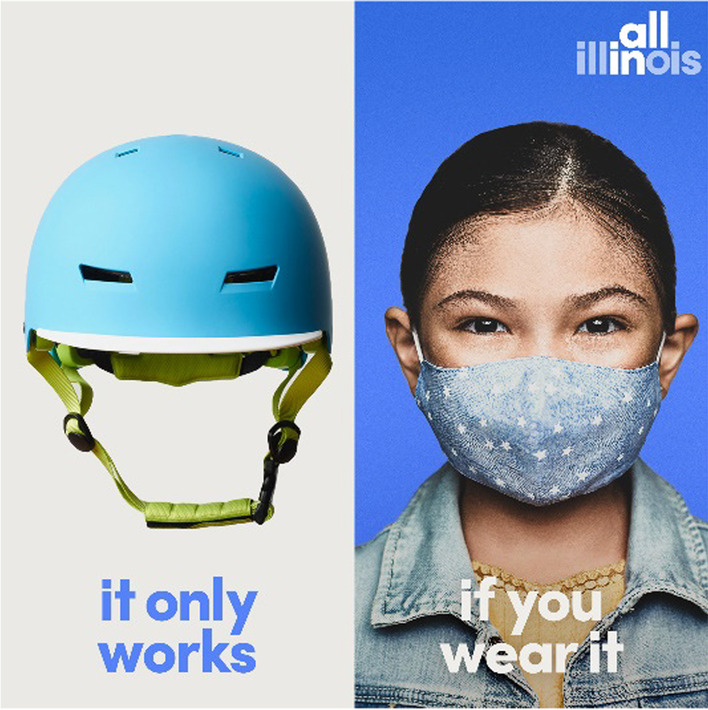
In the United States, an ad campaign compared masks to helmets and seatbelts. The campaign, which was initiated after wearing a mask had been made mandatory in most public places but only 66% of residents said they always wore a mask, was based on a survey that showed this was an effective message

The principles in Table 2 could also be applied to decisions about whether to restrict people’s behaviour or mandate that people behave in a certain way. When a behaviour is mandated, messages may still be designed to persuade people to adhere to the mandate, or they may be designed to inform people. For example, in the context of a mask mandate, messages may be designed primarily to persuade people to adhere to the mandate (e.g. Fig. 3) or to inform people about the rationale for the mandate (e.g. a guide to when and where masks are mandated, why, and how to select and use masks).

## Conclusion

Both persuading people and informing them are reasonable goals for health communication. However, those goals can be in conflict. Decisions to persuade people may be justified, but the basis for those decisions should be transparent, and persuasive messages should not distort the evidence. Key messages should be upfront, using language that is appropriate for targeted audiences. In addition, it should be easy for those who are interested to dig deeper and find more detailed information, including the evidence and the justification for a recommendation or decision [7].

When there is a public health emergency, persuasion may be justified despite important uncertainties about the balance between the potential benefits and harms. However, when there are important uncertainties, they should be acknowledged. Not disclosing uncertainties distorts what is known, inhibits research to reduce important uncertainties, and can undermine public trust in health authorities. When there are important uncertainties about whether it is justified to persuade people, the impacts of persuading people should be evaluated as rigorously as possible.

## Data Availability

Not applicable.
